# Comparative analysis between the gold standard titration method and a mathematical formula to predict CPAP pressure

**DOI:** 10.1016/j.bjorl.2025.101656

**Published:** 2025-05-29

**Authors:** Cíntia Felicio Adriano Rosa, Adriane Iurck Zonato, Ordival Augusto Rosa

**Affiliations:** Hospital IPO, Curitiba, PR, Brazil

**Keywords:** CPAP, Titration, Mask, Nasal pillow, Sleep apnea, Predictive formulas

## Abstract

**Objective:**

One lower-cost alternative to either manual or APAP titration is predicting therapeutic pressure by using mathematical formulas. This study aimed to determine whether the Miljeteig and Hoffstein predictor formula was equivalent to the pressure established by manual titration in a sleep laboratory in patients using nasal or pillow masks.

**Methods:**

The authors analyzed all CPAP titration polysomnography studies in adult patients for three consecutive months (May 20th to August 19th, 2018) in a sleep-disorders clinic. CPAP pressures were manually titrated over the night. Anthropometric data (age, sex, Body Mass Index [BMI], Neck [NC] and waist circumference) and polysomnographic data were documented. We tested Miljeteig and Hoffstein model formula-predicted CPAP (*Hpred*) accuracy in our patients, comparing formula results to our manual titration; *Hpred* = (0.16 *BMI*) + (0.13 *NC*) + (0.04 *AHI*) - 5.12.

**Results:**

Nasal masks were used in 55% of our patients (n = 86) and pillow masks in 45% (n = 71). There was no difference between groups for age, body mass index, neck circumference, waist circumference, gender, baseline apnea-hypopnea index, CPAP pressure and residual AHI during titration polysomnography. The mean difference obtained between CPAP pressure and *Hpred* formula was 2.4 cm H_2_O. The CPAP pressure means during titration was 2.4 cm H_2_O higher than pressure obtained by a formula for pillow group and 2.3 for the nasal group.

**Conclusions:**

In most patients, formula underestimated the CPAP pressure obtained during titration for both nasal and pillow masks. Even though the pressure is underestimated, this approach could help to define minimum and maximum titration pressure using APAP in patients with OSA until the optimal pressure can be determined in the sleep laboratory if necessary.

**Level of evidence:**

Level 3.

## Highlights

• The high demand leads to the need to explore predictive formulas for CPAP pressure.
• Baseline AHI, BMI, and NC predict higher CPAP pressure during titration.
• Formula could enhance home treatment defining min‒max pressures with APAP.
• Formula underestimated CPAP pressure; reassess if symptoms persist or treatment fails.

## Introduction

Obstructive Sleep Apnea (OSA) is a common health problem, and the prevalence has increased alarmingly over time, mainly due to obesity.^1^, ^2^ A prevalence of 32% in adult patients was found in São Paulo city population.^3^

OSA is an independent risk factor for several disorders and is an important emerging health condition.^2^ The morbidity and mortality associated with this disease emphasize the need for effective treatment.^4^ Continuous Positive Pressure device (CPAP) is the gold standard treatment for moderate to severe OSA, significantly reducing OSA symptoms and their cardiovascular consequences.^5^, ^6^

CPAP therapeutic pressure is defined as the lowest pressure that eliminates apneas, hypopneas, snoring, and sleep fragmentation during the night and could be manually titrated in sleep laboratory.^7^ Auto titration with Automatic Positive Pressure device (APAP) should be considered as an alternative to manual titration in laboratory, due to high prevalence of OSA in the population and the scarcity of polysomnography laboratories, especially during COVID-19 pandemic. This APAP titration should be performed in carefully selected OSA patients without associated risk factors.^8^, ^9^ One lower-cost alternative to either manual or APAP titration is predicting therapeutic pressure by using mathematical formulas.^10^

Several studies showed a variable correlation between the pressure defined by the formula and the one suggested by laboratory titration.^11^ Our previous study showed that pillow masks were as efficient as nasal masks during CPAP manual titration exams.^12^ The objective of present study was to determine whether the Miljeteig and Hoffstein predictor formula was equivalent to the pressure established by manual titration in a sleep laboratory comparing two groups of masks: nasal and pillow.

## Methods

### Study design and subjects

For the present study, we selected data from individuals with OSA who were submitted to CPAP titration from May 20th to August 19th, 2018, as part of our clinical activities at the sleep center of IPO Hospital, Curitiba, Brazil. The original aim of the cohort was to investigate the effectiveness of pillow and nasal masks, as previously described by our research team.^12^

For all patients, the following data were recorded: age, gender, Body Mass Index (BMI), Neck Circumference (NC), Waist Circumference (WC) and baseline Apnea-Hypopnea Index (AHI). The inclusion criteria included age ≥18-years who used a nasal pillow (AirFitP10/ResMed®) or nasal mask (AirFitN20/ResMed®). The exclusion criteria were patients who required bi-level positive airway pressure; submitted to automatic titration; submitted to split-night study, used their mask; used oronasal mask; switched the mask during titration; patients without baseline AHI; a patient who gave up the titration; with no information by technician regarding the mask model and in case of a technical mistake.

### Polysomnography

All patients underwent a full-night CPAP titration polysomnography study according to American Academy of Sleep Medicine (AASM) Standards. Multi-channel recordings of the electroencephalogram (frontal, central, and occipital), electrooculogram (EOG), electromyogram (EMG), oronasal flow (by mask), respiratory effort (by abdominal and thoracic strain gauges), oxygen saturation (pulse oximetry), snoring, and body position were recorded on a computerized workstation (Alice 5, Respironics). Patients used the Philips-Respironics REMstar device. Heated humidification was provided to all patients in the study. A sleep technician manually titrated the CPAP pressure. All titration studies were reviewed by a board-certified sleep medicine specialist. The final prescribed CPAP level for each study was determined by the physician. Effective pressure setting was assumed when apneas, hypopneas and snoring were eliminated.

### Mask choice

The attending technician provided instructions and performed a mask fitting session in all patients. All patients used the same model of nasal mask (AirFitN20/ResMed®) or nasal pillow mask (AirFitP10/ResMed®). Two different masks were presented, and patients were assisted in trying both types of interface – standard nasal mask and nasal pillows. After that, patients were asked to choose which mask they preferred.

Our clinical trial was approved by the Ethical Review Board of our institution. The study was not supported by any sponsor and the authors have no conflict of interest.

### CPAP prediction formula

The Miljeteig and Hoffstein model was the first and most widely known predictive model developed to set CPAP therapeutic pressure.^10^, ^11^

CPAP prediction formula pressure is calculated according to the following equation: Hpred=(0.16BMI)+(0.13NC)+(0.04AHI)–5.12.

Abbreviations: BMI, Body Mass Index; NC, Neck Circumference; AHI, Baseline AHI; *Hpred*, Miljeteig and Hoffstein model.

### Statistical analysis

Data are expressed as mean ± standard deviation, medians, minimum and maximum values. For categorical variables, frequencies and percentages were presented. For the comparison of two groups concerning the baseline AHI, Student's *t*-test for independent samples was used. For continuous quantitative variables that met the normality condition, comparisons were made using the covariance analysis model (ANCOVA) including baseline AHI as the covariate. Other variables were analyzed considering the nonparametric Mann-Whitney test. Categorical variables were analyzed using the chi-square test. To evaluate the correlation between CPAP pressure and FORM pressure variables, Pearson linear correlation coefficients were estimated. For multivariate analysis of factors associated with CPAP pressure, a multiple linear regression model was adjusted. The agreement between two pressure assessment methods was analyzed considering the Bland-Altman method. Data from continuous quantitative variables that did not meet the normal condition were submitted to logarithmic transformation. Values of *p* < 0.05 indicated statistical significance. Statistical analysis was performed using Stata/SE v.14.1. StataCorpLP, USA.

## Results

Data were obtained from 212 consecutive patients underwent a full-night CPAP titration polysomnography. Fifty-five patients were excluded in the analyses: patients who required bi-level positive airway pressure (n = 07); submitted to automatic titration (n = 02); submitted to split-night study (n = 18), used their own mask (n = 04); used oronasal mask (n = 07); switched the mask during the titration (n = 08); patients without baseline AHI (n = 05); patient who gave up the titration (n = 01); with no information regarding the mask model (n = 2) and technical mistake (n = 01). Eight patients (3.7%) changed their initial mask: four of those had chosen nasal pillows and four had chosen standard nasal masks. Finally, we analyzed data from 157 patients. Nasal masks were used in 55% (n = 86) and nasal pillow masks in 45% (n = 71).

The study group characteristics data are shown in Table 1, Table 2. There was no difference between groups for age, body mass index, neck circumference, waist circumference, gender, baseline apnea-hypopnea index, CPAP pressure and residual AHI during titration polysomnography. The residual apnea-hypopnea index was adequate for both groups.

**Table 1:** Patients' characteristics according to the mask used.

	Group	n	Mean	Median	Minimum	Maximum	SD	p^a^
Age (years)	Nasal	86	54.3	56	26	81	12.6	
	Pillow	71	54.1	55	23	88	12.3	0.910
BMI	Nasal	86	30.3	29.8	21.4	43.8	4.5	
	Pillow	71	30.3	29.4	22.3	43.2	4.6	0.906
WC	Nasal	86	105.1	104	84	146	11.5	
	Pillow	71	103.5	104	83	131	12.6	0.418
NC	Nasal	86	41.3	41	33	56	4.1	
	Pillow	71	40.5	41	33	57	4.7	0.254
AHI/Baseline	Nasal	86	49.5	46.1	5.9	110.4	26.0	
	Pillow	71	41.5	34.6	5.5	108	24.5	0.064
CPAP pressure level (cm H_2_O)	Nasal	86	9.4	9	6	14	1.8	
	Pillow	71	9.1	8.5	6	15	2.0	0.610
AHI/residual with CPAP	Nasal	86	3.05	2	0	14.2	2.88	
	Pillow	71	3.55	2	0	23	4.12	0.276

BMI, Body Mass Index; NC, Neck Circumference, WC, Waist Circumference. The mean CPAP level was 9.4 ± 1.8 cm H_2_O for the nasal mask (pressure range was 6–14 cm H_2_O) and 9.1 ± 2.0 cm H_2_O for nasal pillow mask (pressure range was 6–15 cm H_2_O). AHI, Apnea/Hypopnea Index; CPAP, Continuous Positive Airway Pressure.

a Student's *t*-test for independent samples or nonparametric Mann-Whitney test, *p* < 0.05. Data are presented as mean ± standard deviation.

**Table 2:** Gender distribution according to mask.

Gender	Mask	
	Nasal	Pillow
Male	56	39
	65.1%	54.9%
Female	30	32
	34.9%	45.1%
Total	86	71

*p* = 0.194 (Chi-Square test, *p* < 0.05).

**Fig. 1:**
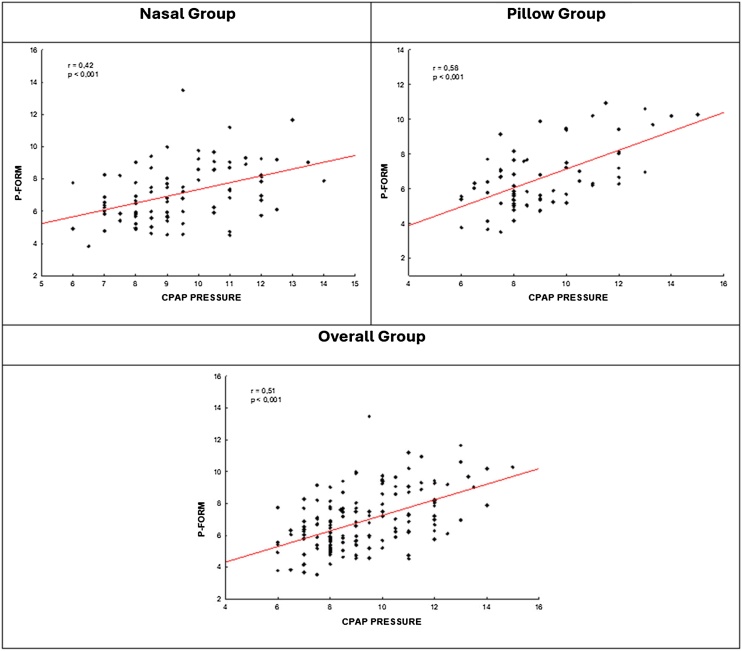
Relationship between P-Form and CPAP pressure. Pearson linear correlation coefficient.

**Fig. 2:**
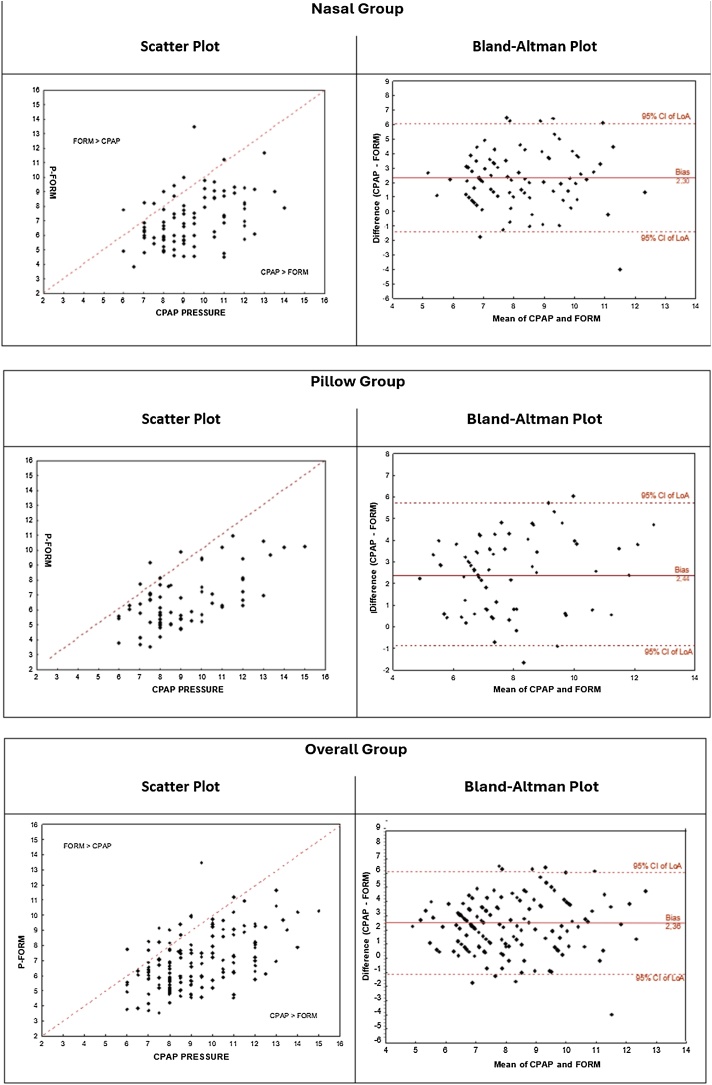
Concordance analysis between CPAP pressure and pressure from Formula (P-Form).

There was a moderate correlation between CPAP pressure and formula pressure (FORM Pressure) (Fig. 1). Concordance analysis revealed that the mean difference between the two measurements is 2.4. The CPAP pressure means during titration was 2.4 cm higher than the pressure obtained by the formula for pillow group and 2.3 for nasal group (Table 3, Table 4). In most patients, formula underestimated the CPAP pressure obtained during titration in both groups and the bias is consistent for lower or higher values of the measurement of CPAP pressure (Fig. 2).

**Table 3:** Concordance analysis between CPAP pressure and pressure from Formula (P-Form).

	n	Mean of differences CPAP-FORM (bias)	Inf lim 95%	Sup lim 95%	*p*^a^
Nasal	86	2.3	−1.5	6.1	<0.001
Pillow	71	2.4	−0.9	5.8	<0.001
Total	157	2.4	−1.3	6.0	<0.001

a Student's *t*-test for paired samples, *p* < 0.05.

**Table 4:** CPAP pressure and pressure from Formula (P-Form).

	Group	n	Mean	Median	Minimum	Maximum	SD	p*
CPAP pressure (cm H_2_O)	Nasal	86	9.4	9	6	14	1.8	
	Pillow	71	9.1	8.5	6	15	2.0	0.610
FORM Pressure (cm H_2_O)	Nasal	86	7.1	6.8	3.8	13.5	1.8	
	Pillow	71	6.6	6.3	3.5	10.9	1.8	0.746

## Discussion

CPAP is a highly effective treatment for OSA.^4^ Due to the cost of CPAP titration tests and high demand of sleep laboratories, mathematical formulas have been explored to predict effective CPAP pressure.^10^, ^11^, ^13^, ^14^, ^15^, ^16^, ^17^, ^18^, ^19^ The objective of present study was to determine whether the Miljeteig and Hoffstein predictor formula was equivalent to the pressure established by manual titration in a sleep laboratory comparing two groups of masks: nasal and pillow.

We found that CPAP titration pressure was 2.4 cm higher than pressure obtained by the formula for the pillow group and 2.3 for the nasal group.

According to a multicenter study on different methods for CPAP titration, there was no statistical difference in adherence to CPAP treatment among home auto titration, titration in a sleep laboratory or use of a mathematical formula to predictive treatment pressure.^11^ A previous work shows that there was no difference in the response of excessive daytime sleepiness (using the Epworth Sleepiness Scale) between groups treated with pressure calculated through a formula or through self-titration: both benefited from a significant decrease in sleepiness.^16^

A systematic review of formulas to predict CPAP therapeutic pressure showed that BMI and mean oxyhemoglobin saturation were the variables with the most impact.^11^ Our group also found that baseline apnea-hypopnea index, BMI, and NC were independent predictors of a higher CPAP pressure during titration. Moreover, we found Waist Circumference (WC) also had statistical significance (<0.001).^12^ Miljteig and Hoffstein showed OSA severity (AHI), obesity (BMI) and Neck Circumference (NC) were the most important factors to predict CPAP pressure. The authors suggested the following formula: CPAPform=(0.16BMI)+(0.13NC)+(0.04AHI)–5.12.^10^

Despite CPAP devices can be titrated, one study reviewed the literature to justify the use of a standard fixed therapeutic CPAP pressure as the initial setting. The authors proposed CPAP should be initially prescribed at 9 cmH_2_O to all recently diagnosed OSA patients who would normally be eligible for this treatment.^19^ In our study the mean CPAP titration laboratory level was 9.4 ± 1.8 cm H_2_O for the nasal mask and 9.1 ± 2.0 cm H_2_O for nasal pillow. A prospective, randomized, crossover, single-blinded study analyzed 46 patients conclude that fixed CPAP pressure may be considered as initial therapy based on subjects' preference for moderate and severe OSA if titrated CPAP pressure less than 8 cm H_2_O and BMI less than 32.3 kg/m^2^.^20^

In most patients, formula underestimated the CPAP pressure obtained during titration, as already observed in other studies.^11^ Gokcebay showed that when CPAP pressure is higher than 10 cm H_2_O, this formula usually underestimated the titration pressure, but in our study, formula also underestimated the CPAP pressure, and the mean CPAP level was lower than Gokcebay’s study.^21^ In a study in the Asian population, the use of a predictive formula based on BMI, minimum oxygen saturation, Respiratory Disorder Index (RDI), and Epworth Sleepiness Scale (ESS) was effective in establishing the CPAP pressure in 38.8% of the patients against 36.5% of the Miljteig formula.^14^ Schiza tested predictive formulas in the Greek population and found an efficacy of 79% with the Miljteig formula, and 95% with a formula using BMI, gender, smoking, and Apnea-Hypopnea iIndex (AHI).^15^

The pressure at or below which the APAP device spends 90% or 95% (90th or 95th percentile) of usage is usually the pressure chosen if a patient will use a fixed-pressure CPAP therapy.^22^ A retrospective study included 49 patients with OSA comparing CPAP pressure using APAP (defined as 95th percentiles pressure) and pressure calculated according to Body Mass Index (BMI), Neck Circumference (NC), and Apnea/Hypopnea Index (AHI) formula. The mean in APAP group (10.4 ± 1.8 cm H_2_O) was significantly higher than the mean formula group (7.82 ± 1.51 cm H_2_O).^23^ In our study, CPAP titration pressure in laboratory was also higher than pressure obtained by the formula. Another study showed that the formula could be used to help define minimum and maximum pressure using APAP. The authors found no statistical difference in both adherence and AHI improvement among three groups: one group using fixed CPAP with pressure by titration in a sleep laboratory, the second using APAP with the pressure interval based on titration laboratory pressure and other group using APAP with the pressure interval based on the Miljteig formula.^24^

Higher than needed PAP pressures may indirectly add to poor adherence through dryness, pressure discomfort and development of treatment-emergent central sleep apnea in some patients.^21^ One of the limiting factors to become CPAP effective is adherence to treatment. This adherence varies according to the parameters used in the studies, and results between 29% and 83% of adherence of patients that use an adequate number of >4 h per night of CPAP use.^25^ Pressure discomfort is one of the main contributors to non-adherence to CPAP.^26^ Therefore, adjusting the CPAP pressure earlier during the initial treatment of the patient with OSAS may be facilitate patient adherence to treatment.

More studies are needed with greater analysis of variables, checking possible phenotypes to predict a CPAP treatment pressure more accurately through mathematical formulas and it use for prescribing CPAP pressure for home. However, the use of predetermined pressures through formulas could be used to optimize the CPAP titration study by increasing the starting pressure of the titration, especially split-night type.^11^, ^14^, ^27^

The formula could be a rational choice that facilitates faster and easier cost-saving access to CPAP therapy. Nevertheless, a reassessment and, perhaps, a CPAP titration study should be done in case the patient still manifests residual symptoms or if treatment appears to lack efficacy. This is particularly important during the initial of using CPAP.^8^

### Limitations of the study

The study design may have introduced bias due to the non-randomization of mask type. Moreover, it did not randomly assign the two interfaces for many reasons. First, it wanted to assess patients’ mask preference. Second, it was believed that allowing patients to use a mask of their choice would be an important motivator. Patients included in this study could change their mask type if their initial choice did not meet their expectations before the exam started. We excluded patients who required bi-level positive airway pressure, submitted to automatic titration and/or submitted to split-night that affected the generalizability of the findings to this groups of patients. Despite the exclusion of patients from the study, the sample still comprises 157 patients who underwent the gold standard CPAP titration test. Finally, one strength of the research is that the same model of nasal mask or pillow was used, which reduces detection bias within each group.

## Conclusion

The present study shows that the formula underestimated the CPAP pressure obtained during titration for both nasal and pillow masks in 2.4 cm H_2_O on average. Even though the pressure is underestimated, this approach could help to define minimum and maximum titration pressure using APAP in patients with OSA until the optimal pressure can be determined in the sleep laboratory if necessary.

## Credit authorship contribution statement

CFAR and AIZ participated in data collection and manuscript writing; OAR participated in critical manuscript revision. All authors had substantial contributions to drafting the article or revising it critically for important intellectual content and approved the final version of this article.

## Ethics approval

All procedures performed in studies involving human participants were following the ethical standards of the institutional and/or national research committee and with the 1964 Helsinki declaration and its later amendments or comparable ethical standards. Informed consent was obtained and was approved by the Ethical Review Board of Hospital IPO, city of Curitiba‒Paraná Brazil.

## Code availability

Not applicable.

## Funding

This study received no funding.

## Declaration of competing interest

All authors have no actual or potential conflicts of interest to disclose, including financial interests and relationships.
